# Cryptogenic Pontine Abscess Treated With Stereotactic Aspiration: A Case Report

**DOI:** 10.7759/cureus.41463

**Published:** 2023-07-06

**Authors:** Taylor M Ngo, Anna Okabe, Kailey B Nguyen, Anhtho Tong, Jason Chang, Forshing Lui

**Affiliations:** 1 Neurology, California Northstate University College of Medicine, Elk Grove, USA; 2 Neurology, Kaiser Permanente South Sacramento Medical Center, Sacramento, USA; 3 Clinical Sciences, California Northstate University College of Medicine, Elk Grove, USA

**Keywords:** pontine abscess, idiopathic brain abscess, stereotactic needle aspiration, brainstem abscess, brain abscess

## Abstract

Brainstem abscesses are localized collections of pus or infected material within the brainstem, which can cause inflammation, tissue damage, and compression of adjacent structures. This can lead to a variety of symptoms, including headache, fever, and focal neurological deficits, among many others. Brainstem abscesses are potentially life-threatening and considered to be rare, and pontine abscesses are even rarer. Both are often caused by the spread of infection from nearby structures like the middle ear, sinuses, and mastoid air cells, but they can also result from distant infectious sites that have spread to the bloodstream. Ambiguous clinical presentation can delay appropriate care and lead to poorer outcomes. We present a rare case of pontine abscess in a 54-year-old male with both undetermined causal origins and unclear infectious signs, namely, the lack of fever, fatigue, and chills. We will discuss the etiologies, diagnosis, and treatment of cryptogenic brainstem lesions in this case report.

## Introduction

Brain abscesses are rare yet life-threatening infections of the parenchyma, with an incidence rate ranging from 0.3 to 1.3 cases per 100,000 individuals per year [1]. They are collections of pus caused by diverse microorganisms. Most intracranial abscesses are localized to the cerebrum, particularly in the frontal and temporal lobes, primarily due to the spread of adjacent infections in the paranasal sinuses and middle ear caused by oropharyngeal organisms [2]. Abscesses located in the brainstem are even more rare, accounting for less than 1% of brain abscess cases, and are due to the hematogenous spread of organisms [1]. When considering brainstem abscesses, the most common location is in the pons in both adult and pediatric populations [3,4]. Patients most commonly present with symptoms similar to meningitis, including fever, headache, and focal neurological symptoms, and the absence of fever can make clinical diagnosis much harder. Further, the symptoms and subsequent management can vary based on the location and size of the abscess as well as the underlying microorganism.

The site of primary infection along with the patient’s age and immune status are important factors that influence the susceptibility to certain pathogens. For instance, immunocompromised patients are more at risk for fungal infections, whereas immunocompetent individuals are more likely to be infected with bacterial pathogens [5]. *Streptococcus* and *Staphylococcus* species have been identified as the most frequent causes of brain abscesses, followed by anaerobic and gram-negative bacteria [2]. The majority of patients with brain abscesses have predisposing conditions, such as ear or nasal infections, recent surgical procedures, penetrating trauma, or other sources that can cause direct spread from a contiguous site. Hematogenous spread from distal infections, including infective endocarditis, can also introduce pyogenic pathogens within the brain [4]. We present a rare case of pontine abscess that posed diagnostic challenges due to the absence of typical symptoms associated with brain abscess.

## Case presentation

**Figure 1:**
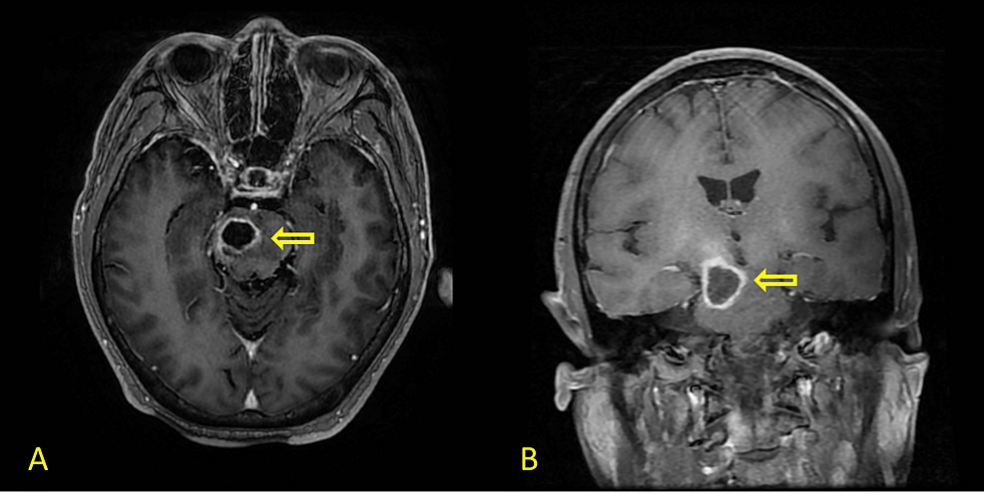
Contrast-enhanced head MRI showing ring-enhancing lesion in right pons (yellow arrow) in both axial (A) and coronal (B) views.

A 54-year-old Vietnamese male presented with numbness and clumsiness in his left hand and leg, which developed over a three-day period. These symptoms were preceded by a headache that awoke him the night before without any accompanying fever. Neurologic examination upon initial presentation revealed full orientation and alertness, normal speech and language, and unimpaired cranial nerve function. Motor testing was notable for 4/5 left finger extension, 4/5 left ankle dorsiflexion, and left pronator drift, while cerebellar testing was notable for left heel-to-shin dysmetria. Deep tendon reflex testing showed 1+ grading for the left bicep and 3+ grading for both patellae. The initial brain magnetic resonance imaging (MRI) showed a 1.5 cm ring-enhancing lesion localized to the right pons (Figure 1). A complete blood count (CBC) on admission showed a white blood cell (WBC) count of 4.2, platelet count of 147, hemoglobin (Hb) of 11.9, and hematocrit (Hct) of 36.6. The patient’s blood cultures were negative as were screenings for methicillin-resistant *Staphylococcus aureus* (MRSA), human immunodeficiency virus (HIV), and hepatitis B virus (HBV). Cysticercosis antibody testing was equivocal, while an interferon-gamma release assay was negative. The patient was started on ceftriaxone, vancomycin, and metronidazole.

**Figure 2:**
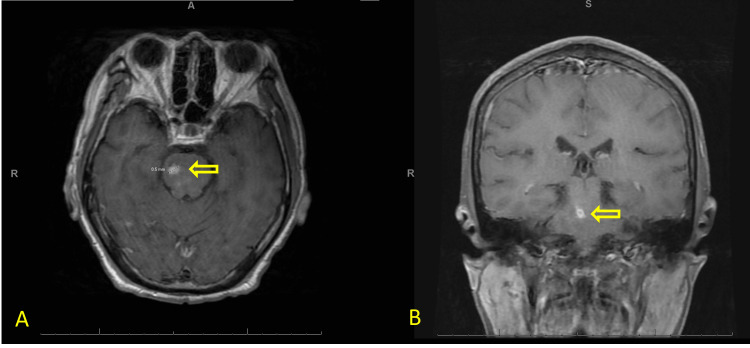
Postoperative head MRI with T2 fluid-attenuated inversion recovery sequence showing reduced ring-enhancing lesion in right pons (yellow arrow) in both axial (A) and coronal (B) views.

Three days later, the patient showed progressively worsening weakness in his left arm and leg, new onset of left-sided facial tingling, and binocular diplopia. Neurologic examination showed significant left upper extremity (LUE) plegia, with motor testing revealing 2/5 elbow flexion, 1/5 elbow extension, 1/5 wrist flexion, 0/5 wrist extension, 3/5 finger flexion, and 1/5 finger extension. The patient also displayed dysarthria and right gaze palsy. A repeat MRI was done, which showed a thickening of the enhancing wall and the formation of another lesion in the right dorsal paramedian pons. A lumbar puncture was then performed, which showed 11 WBCs, two red blood cells (RBCs), a glucose level of 76 mg/dL, and a protein level of 62 mg/dL. Cerebrospinal fluid (CSF) was clear, colorless, and negative for cultures, toxoplasma IgG, cryptococcal antigen, herpes simplex virus (HSV), and acid-fast bacteria. Dexamethasone was added to his empiric treatment. He was then transferred to another hospital for transfrontal stereotactic drainage of the lesion, which was performed using the standard deep brain stimulation (DBS) entry point of the coronal suture. Drainage of the abscess revealed 1.3 cc of a purulent, foul-smelling material with a yellow-green-gray consistency, and the cultures of this material grew *Streptococcus pyogenes* (group A Strep). A postoperative MRI showed a decrease in the size of the abscess to 0.85 cm without hemorrhage (Figure 2). The patient stated improvements in his strength and functionality, although there was still some residual hemiparesis with strength testing showing 3/5 proximal LUE, 3/5 hand grip, and 4/5 left lower extremity (LLE) grading. An eight-week course of ceftriaxone, vancomycin, and metronidazole was to be continued following the needle aspiration, and vancomycin and ceftriaxone levels were monitored through minimum inhibitory concentration.

Five days following the aspiration, the patient had labs drawn at the hospital where he received surgery. Comprehensive metabolic panel (CMP) results included a sodium level of 134 mEq/L, glucose level of 208 mg/dL, blood urea nitrogen (BUN) of 26 mg/dL, calcium level of 7.0 mg/dL, and elevated liver function tests (LFTs). His CBC showed a WBC count of 7.5, platelet count of 115, Hb of 10.8, and Hct of 32.7. These results suggested developing pancytopenia, which could be attributed to vancomycin use, so the medication was discontinued. A month after the aspiration, when the patient was seen at the home hospital, he still exhibited persistent pancytopenia. His CBC revealed a WBC count of 3.2, platelet count of 137, Hb of 9.5, and Hct of 27. These results indicated that ceftriaxone was causing the patient’s pancytopenia, so it was discontinued while vancomycin was re-added to his drug regimen. Following eight weeks of antibiotic treatment, all medications were stopped after verification of decreasing abscess size on a follow-up MRI.

## Discussion

Brain abscesses are rare, but brainstem abscesses are even more rare, accounting for less than 1% of intracranial abscesses [2]. In a retrospective study of 973 patients with brain abscesses conducted by Nathoo et al. (2011), it was determined that only three of these patients had abscesses located in the brainstem [6]. Therefore, the literature surrounding the etiology and prognosis of adult brainstem abscesses is greatly lacking.

Patients with brain abscesses commonly present with a triad of fever, headache, and nausea. In the study conducted by Nathoo et al. (2011), they found that only 4.6% of cases had cryptogenic etiologies [6]. In these cases, patients have insidious causes of infection and present with no clear history or symptoms of infection, which can greatly hinder the time to a proper diagnosis. In another study conducted by Helweg-Larsen et al. (2012), 39% had no fever, 26% had normal C-reactive protein, and 49% had no leukocytosis [7]. Therefore, there are a minority of patients with brain abscesses lacking signs indicative of infection. While our patient had headaches prior to the neurologic deficits, he did not present with any signs of fever. Another point of interest in our case was the fact that the stereotactic aspiration cultures grew *Streptococcus pyogenes* (group A Strep), which is usually a skin flora but may exist in the oropharynx. Yet, our patient denied recent upper respiratory illness. Some streptococcal species, including *Streptococcus* *bovis*, are associated with bowel disease, but this is a highly unlikely cause due to the unusual location of the lesion [8].

Early diagnosis and initiation of treatment are critical to prevent adverse outcomes following brain abscesses, such as permanent neurological damage. Diagnostic delay is known to be a major contributor to the severity of outcomes [9]. However, the nonspecific symptoms and clinical presentations without clear signs of infection pose a great barrier to prompt management. The current gold standard for diagnosis of brain abscesses is through neuroimaging, such as a contrast-enhanced MRI scan. CT scans are helpful for the localization and identification of the number of abscesses but do not allow for the differentiation between an abscess and a tumor. Therefore, an initial CT without contrast is a poor choice of imaging and can result in diagnostic delays. In the study conducted by Helweg-Larsen et al. (2012), 10 patients underwent initial CTs, which were interpreted as a tumor or stroke [7]. MRIs are a more sensitive tool for the diagnosis of brain abscesses, and diffusion-weighted MRIs have been shown to have a sensitivity and specificity of 96% for brain abscesses [10]. Imaging of our patient revealed the characteristic ring-enhancing lesion. Although a definitive diagnosis of brain abscess cannot be made until surgery, it is important to initiate neuroimaging early upon suspicion of an abscess for prompt intervention.

Treatment of solitary brainstem abscesses typically involves a combination of antimicrobial therapy and surgical intervention. The treatment approach varies depending on the underlying cause of the abscess as well as the location and size of the lesion. For brainstem abscesses of undetermined origins and where the clinical diagnosis can be challenging, the best mode of treatment remains unknown. Previous literature calls for a conservative strategy in which abscesses measuring less than 2.5 cm are treated solely through broad-spectrum antibiotics [11]. For abscesses larger than 2.5 cm, neurosurgery by stereotactic aspiration or microsurgery is recommended both for the purposes of diagnosis and decompression [12]. However, it has been suggested that nearly all brain abscesses measuring at least 1 cm may be subject to stereotactic aspiration, regardless of the location [13]. Therefore, we emphasize the value and utility that stereotactic aspiration, a minimally invasive procedure, can have in cases of cryptogenic brainstem abscesses, as echoed by Hamamoto et al. (2011) [14]. It can assist in the prompt isolation and identification of causal pathogens, leading to the correct antimicrobial drug choice and optimal treatment.

## Conclusions

We present a rare case of a patient with a cryptogenic brainstem abscess located in the pons who was successfully treated with transfrontal stereotactic needle aspiration and an antibiotic regimen. Given the crucial role of the pons and rarity of this condition, it is important to showcase novel cases, such as ours, where successful treatment has been achieved. This will contribute valuable insights into the potential management of this disease. Additionally, the nonspecific clinical presentation of brainstem abscesses may lead to delayed intervention and poorer outcomes. We stress the early identification of organisms involved to initiate early treatment.
